# The Role of the Central Nervous System Microenvironment in Pediatric Acute Lymphoblastic Leukemia

**DOI:** 10.3389/fped.2017.00090

**Published:** 2017-04-26

**Authors:** Nathan P. Gossai, Peter M. Gordon

**Affiliations:** ^1^Division of Pediatric Hematology and Oncology, University of Minnesota, Minneapolis, MN, USA; ^2^University of Minnesota Masonic Cancer Center, Minneapolis, MN, USA

**Keywords:** acute lymphoblastic leukemia, central nervous system, chemoresistance, migration, microenvironment, niche

## Abstract

Acute lymphoblastic leukemia (ALL) is the most common cancer in children. While survival rates for ALL have improved, central nervous system (CNS) relapse remains a significant cause of treatment failure and treatment-related morbidity. Accordingly, there is a need to identify more efficacious and less toxic CNS-directed leukemia therapies. Extensive research has demonstrated a critical role of the bone marrow (BM) microenvironment in leukemia development, maintenance, and chemoresistance. Moreover, therapies to disrupt mechanisms of BM microenvironment-mediated leukemia survival and chemoresistance represent new, promising approaches to cancer therapy. However, in direct contrast to the extensive knowledge of the BM microenvironment, the unique attributes of the CNS microenvironment that serve to make it a leukemia reservoir are not yet elucidated. Recent work has begun to define both the mechanisms by which leukemia cells migrate into the CNS and how components of the CNS influence leukemia biology to enhance survival, chemoresistance, and ultimately relapse. In addition to providing new insight into CNS relapse and leukemia biology, this area of investigation will potentially identify targetable mechanisms of leukemia chemoresistance and self-renewal unique to the CNS environment that will enhance both the durability and quality of the cure for ALL patients.

## Introduction

Acute lymphoblastic leukemia (ALL) represents ~25% of all pediatric cancer diagnoses and, despite significant advances in therapy, it is still a common cause of death in children with cancer (1). While leukemia arises in the bone marrow (BM), it is a systemic disease with a predilection for certain organs such as the central nervous system (CNS). Prior to the advent of CNS-directed leukemia therapies, CNS leukemia developed in over half of pediatric leukemia patients (2–4). Moreover, despite current CNS-directed therapies that often include high-dose systemic chemotherapy, intrathecal chemotherapy, and cranial irradiation in a subset of children, CNS relapse accounts for ~30% of initial relapses in some clinical trials and occurs in ~2–8% of children with leukemia (5–7). Neither the factors that determine the site of leukemia relapse (CNS, BM, other extramedullary, or combined sites) nor why BM relapse confers a significantly worse prognosis than CNS relapse is well understood (8).

Furthermore, sparing cranial irradiation from all but the most high-risk patients, or even all patients in some recent clinical trials (9, 10), requires intensification of the other CNS-directed therapies. Although the elimination of radiation negates the risk of secondary brain tumors and neuroendocrine failure, CNS-directed chemotherapy carries the risk of seizures, encephalopathy, and neurocognitive toxicities that can include significant and persistent impairments in intelligence, processing speed, memory, academics, executive function, and attention (11–18). An extensive literature comprehensively reviews the clinical aspects of CNS leukemia, thus it will not be addressed in this mini-review (5–7, 19).

Rigorous basic science and clinical data demonstrate that leukemia cell-autonomous factors play a critical role in leukemia biology (20–23). The composition and organization of the BM is highly complex with multiple different cell types and soluble factors interacting with and influencing leukemia cells. These elements create distinct niches, which exert unique and functionally important effects on leukemia development, quiescence, maintenance, and chemoresistance (24–33). Accordingly, strategies to disrupt mechanisms of BM microenvironment-mediated chemoresistance or quiescence represent new, promising approaches to cancer therapy (34).

The role of the CNS in regulating leukemia survival and chemoresistance is much less well understood than the BM microenvironment, but is also likely to be important for both understanding leukemia biology as well as developing more effective and less toxic therapies. As the BM and CNS are distinct environments, research investigating the role of the BM in leukemia is unlikely to uniformly translate to the CNS. In this mini-review, we will describe emerging evidence supporting a role for the CNS in regulating critical aspects of leukemia biology and highlight how this area of investigation may translate into more effective and less toxic therapies for patients.

## Approaches for Studying CNS Leukemia

**Figure 1:**
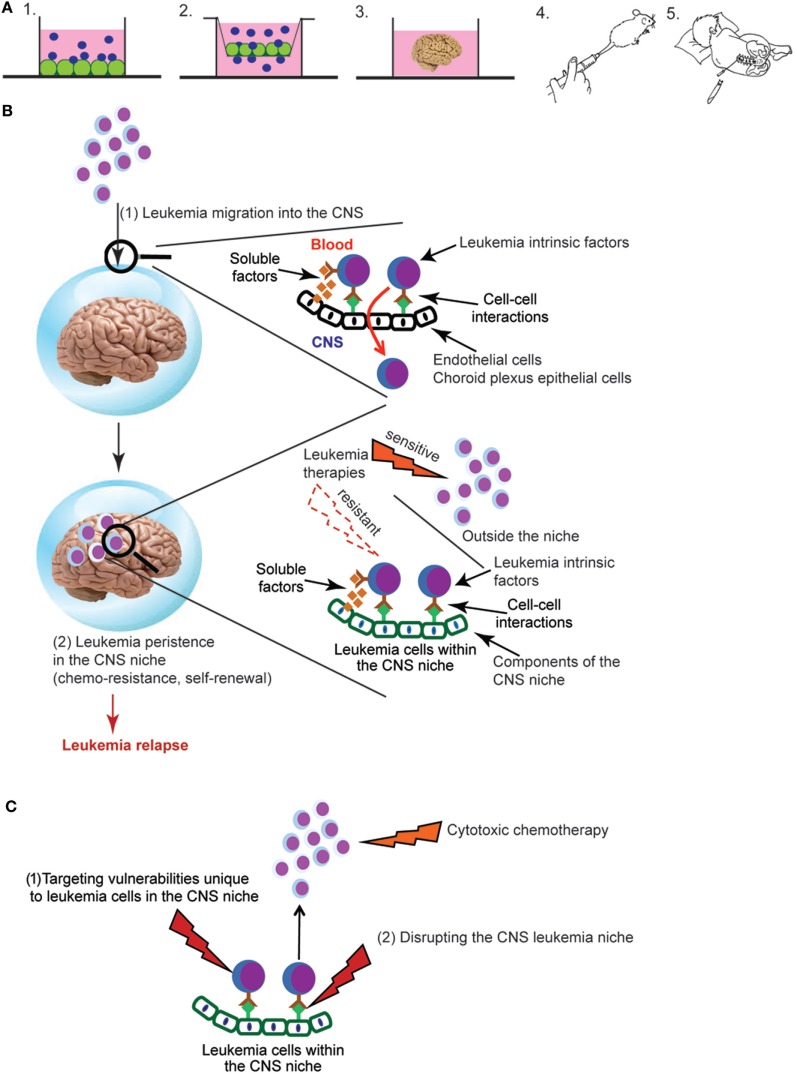
**(A)** Experimental approaches for studying central nervous system (CNS) leukemia. Complementary experimental approaches for studying CNS leukemia include (1) *in vitro* co-culture of leukemia and CNS-derived cells, such as meningeal, glial, or choroid plexus (CP) cells, (2) trans-well migration assays of leukemia cells across either endothelial cells or CP epithelial cells, (3) cerebral organoids that are grown *in vitro* from human induced pluripotent stem cells, (4) *in vivo* murine models that include either genetically engineered mouse models or xenotransplantation of human leukemia cells into immunodeficient mice, or (5) patient-derived leukemia samples isolated from either the bone marrow or cerebral spinal fluid (CSF). Leukemia cells are shown in blue and CNS-derived cells in green. **(B)** Overview of key aspects of the pathophysiology of CNS leukemia and relapse. Leukemia cells first breach the blood–brain and/or blood–CSF barriers, illustrated by the blue sphere (1). Leukemia cells must then persist in the CNS and escape the effects of chemotherapy and immune surveillance in order to lead to relapse (2). Identifying the leukemia cell extrinsic factors (soluble factors, cell–cell interactions) that mediate both of these processes as well as their effects on critical aspects of leukemia biology are active areas of investigation. **(C)** Approaches for targeting leukemia cells in the CNS niche. New therapeutic opportunities will be identified as studies to (i) define the influence of the CNS niche on critical aspects of leukemia biology, such as chemoresistance, self-renewal, and quiescence and (ii) identify CNS-derived factors that protect and maintain leukemia cells in the CNS niche. Directly targeting vulnerabilities unique to leukemia cells in the CNS niche (1) or disrupting the interactions between leukemia cells and the niche (2) represent novel approaches to leukemia therapy.

Defining the role of the CNS microenvironment in leukemia requires experimental approaches for studying and characterizing leukemia cells in the CNS. Fortunately, many previously developed approaches for studying the BM microenvironment in leukemia and normal hematopoiesis have been, or can be, adapted to the CNS microenvironment (Figure 1A). *In vitro* co-culture of leukemia cells with CNS-derived cells can be used to examine the effect of both direct cell–cell interactions as well as soluble factors on leukemia biology (35–37). Similarly, *in vitro* transwell assays that assess the migration of leukemia cells across endothelial or choroid plexus (CP) cells can be used to model the process of leukemia migration across the blood–brain or blood–cerebral spinal fluid (CSF) barriers, respectively (38–41). Cerebral organoids, three-dimensional *in vitro* cultures that model brain organogenesis, represent a new and powerful model system that could also potentially be exploited for expanding our understanding of CNS leukemia (42). Genetically engineered mouse leukemia models and human leukemia xenografts develop CNS leukemia with a frequency and in anatomic patterns that recapitulate human CNS leukemia (43, 44). Accordingly, these *in vivo* systems are powerful tools for biologic discovery as well as therapeutic drug testing. Finally, leukemia cells isolated from the CSF of leukemia patients can be used to confirm and extend knowledge gained in the laboratory. A significant, but not insurmountable, challenge is that only about ~15% of children have morphologic evidence of CNS leukemia at time of diagnosis (45, 46). Moreover, the number of leukemia cells that can be isolated from the typical volume of CSF obtained during a lumbar puncture may be limited. However, with cutting-edge technologies that require only a small number of cells, or single cells, one can envision increasingly sophisticated questions being addressed using primary leukemia cells obtained from CSF samples of newly diagnosed or relapsed patients.

## Trafficking of Leukemia Cells to the CNS

The CNS is an immunologically privileged site that is isolated from the blood system by blood–brain and blood–CSF barriers. Although the process by which normal leukocytes traffic into the CNS has been well studied and reviewed extensively (47–51), this process is less well understood in the context of leukemia. Additionally, tight junctions between epithelial, rather than endothelial, cells provide the blood–CSF barrier within the CP (52). It is not clear that principles governing trans-endothelial migration will apply to the CP. Based on patient autopsy reports and murine leukemia xenotransplantation studies, a rudimentary anatomical and temporal course of leukemia invasion of the CNS can be posited (2, 43). After transiting CP epithelial cells and/or meningeal postcapillary venules, leukemia cells are initially localized to the leptomeninges on the surface of the brain and within the CSF. Leukemia cells then migrate into the deeper meningeal tissues surrounding vessels in the cortex and white matter (Virchow–Robin or perivascular spaces). Only late in the disease is the pial-glial membrane destroyed and leukemia cells identified within the brain parenchyma.

### Leukemia Migration

The contributions of leukemia cell intrinsic and extrinsic factors to CNS trafficking remain incompletely understood (Figure 1B). Yet, an increasing number of leukemia genes and pathways, as well as leukemia cell autonomous factors, have been identified as playing a role in CNS trafficking (Table 1). Not surprisingly, factors implicated in trans-endothelial migration of leukemia cells and leukocytes outside of the CNS also appear to play a role in crossing the blood–brain or blood–CSF barriers. For example, myosin II, a cytoskeleton class II non-muscle myosin motor protein, has been shown to be important for T-cell extravasation (53, 54). Using a pre-B ALL murine leukemia model, Wigton et al. showed that myosin-IIA depletion or inhibition with either shRNA or blebbistatin, respectively, significantly decreased leukemia infiltration into the CNS as a result of impaired trans-endothelial extravasation (55). Similarly, known inhibitors of T-cell leukemia migration diminished the ability of leukemia cells to cross CP epithelial cells in a transwell assay designed to mimic the blood–CSF barrier (38). Using similar *in vitro* co-culture and transwell assays, with brain-derived endothelial cells rather than CP cells, Akers et al. showed VE-cadherin expression by leukemia cells enhanced adhesion to endothelial cells while PECAM-1 expression enhanced adhesion to, and migration through, endothelial cells (39). Together, these studies suggest that many of the cellular mechanisms governing the trans-endothelial migration of leukocytes and leukemia cells outside the CNS are likely to also apply to the transit of leukemia cells across the blood–brain and blood–CSF barriers.

**Table 1:** **Selected genes and pathways that have been associated with central nervous system (CNS) leukemia**.

Gene/pathway/molecule	Putative role	Reference
lnterleukin-15	CNS trafficking	Cario et al. (57)
	Leukemia proliferation	Williams et al. (60)
	NK cell activation	Frishman-Levy et al. (72)
CCR7/CCL19	CNS trafficking of T-cell acute lymphoblastic leukemia (ALL)	Buonamici et al. (44)
VE-cadherin and PECAM1	Adhesion and CNS trafficking	Akers et al. (39)
Asparaginyl endopeptidase, intercellular adhesion molecule 1, ras-related C3 botulinum toxin substrate 2	CNS trafficking	Holland et al. (56)
Mer tyrosine kinase	Chemoresistance and quiescence	Krause et al. (37)
PBX1	Chemoresistance and self-renewal t(1;19) translocation and CNS relapse	Gaynes et al. (36)
		Jeha et al. (68)
VEGFA	Leukemia survival in CNS	Kato et al. (73)
Oxidative phosphorylation	Downregulated in ALL cells in CNS	Kato et al. (73)
SCD, SPP1	CNS trafficking and/or survival	Van der Velden et al. (76)

### Leukemia Genes and Pathways Implicated in CNS Migration

Focusing more specifically on the trafficking of leukemia cells to the CNS, Buonamici et al. used murine T-cell leukemia models involving expression of the oncogenic, intracellular Notch1 fragment in hematopoietic progenitors combined with gene expression profiling to identify the chemokine receptor CCR7 as an essential adhesion molecule required for the infiltration of leukemic T-cells into the CNS (44). Silencing of CCR7 in leukemia cells, or one of its ligands CCL19 in mice, specifically diminished infiltration of the CNS, but not other tissues, in both murine and xenotransplantation leukemia models. The other ligand for CCR7, CCL21, was undetectable in mouse brain sections and presumed to be less important for leukemia migration into the CNS. Interestingly, deletion of CCR7 in two models of B-cell leukemia failed to inhibit CNS infiltration, suggesting that CCR7 function may be specific for Notch1-induced T-cell leukemia. Complementing this genomic approach, Holland et al. used semiquantitative proteomics to compare the plasma membrane protein composition of an invasive pre-B ALL cell line that resulted in CNS leukemia when transplanted into NOD-SCID mice versus two pre-B ALL cell lines with less invasive behaviors (56). Proteins upregulated on the membrane of the more invasive leukemia cell line classified into a number of biological classes likely functionally relevant for leukemia trafficking to the CNS, including cytoskeletal organization, adhesion, migration, invasion, signaling, and endocytosis. Finally, further functional characterization of three of the differentially expressed proteins, asparaginyl endopeptidase, intercellular adhesion molecule 1, and ras-related C3 botulinum toxin substrate 2, suggested a complex role for multiple proteins and cellular processes in the pathogenesis of CNS disease in pre-B-cell ALL.

### Soluble Factors Implicated in CNS Migration

Cytokines and chemokines also likely play a role in the trafficking of leukemia cells to the CNS. Interleukin-15 (IL-15) single-nucleotide polymorphisms and/or mRNA levels have been implicated in leukemia chemoresistance as well as likelihood of CNS disease in leukemia (57–59). While the role for IL-15 in leukemia proliferation and survival is discussed in the next section, IL-15 also upregulates p-selectin glycoprotein ligand-1 (PSGL-1) and CXCR3 levels in leukemia cells (60). Both PSGL-1 and CXCR3 have been implicated in the migration of leukocytes across the blood–CSF barrier and may play a similar role in leukemia (61, 62). However, it is worth noting that other xenotransplantation studies of primary pre-B ALL samples failed to identify a chemokine receptor signature that correlated with CNS invasiveness (43).

## The Role of the CNS Microenvironment in Leukemia Biology

While defining the mechanisms by which leukemia cells traffic from the blood to the CNS is important for understanding the biology and pathophysiology of leukemia, a number of observations may diminish its importance for understanding CNS relapse. First, ~15% of patients show evidence of CNS leukemia by morphological examinations of CSF (45, 46). However, more sensitive examinations of CSF using PCR or flow cytometry detect leukemia cells in up to ~40% of patients at diagnosis (63–66). Second, high rates (~50–75%) of CNS leukemia developed in patients prior to the development of adequate CNS-directed therapies (2–4). Third, ~80% of mice transplanted with human, primary B-cell precursor leukemia cells developed CNS leukemia despite the majority of leukemia samples coming from patients without morphological evidence of CNS leukemia (43). Fourth, clonal tracking of xenotransplanted leukemia cells demonstrated that the composition of leukemia cells in the CNS was polyclonal and similar in composition to the spleen or femur (43). Finally, CNS leukemia relapses occur despite cranial irradiation and/or high-dose systemic and intrathecal chemotherapy that either overcome or bypass the blood–brain barrier, respectively (10). Together, these observations support a model in which the ability of leukemia cells to persist in the CNS and escape the effects of chemotherapy and immune surveillance may contribute more to relapse and therapy resistance than the ability of leukemia cells to migrate from the BM and blood to the CNS (Figure 1B). Further supporting this hypothesis, Akers et al. showed that co-culture of leukemia cells with astrocytes, CP epithelial cells, or meningeal cells enhanced leukemia cell resistance to dexamethasone, cytarabine, and methotrexate-induced cell death (35). Notably, these drugs play an important role in CNS leukemia therapy and prophylaxis. Their work also suggested that both soluble factors secreted by the CNS-derived cells and adhesion-mediated signaling contributed to chemoresistance.

### CNS Relapses in Pre-B ALL with t(1;19) Translocation

Mechanisms of leukemia chemoresistance in the CNS have also been studied in the context of leukemia bearing the t(1;19) translocation. This translocation occurs in ~5% of pre-B ALL, is associated with an increased risk for CNS relapse, and has been associated with increased expression of the Mer receptor kinase (37, 67, 68). Mer kinase is involved in multiple physiological processes including cell survival, migration, and differentiation (69). Its overexpression or ectopic expression has also been implicated in a wide array of cancers. High Mer-expressing t(1;19) leukemia cells co-cultured with CNS-derived cells exhibit G0/G1 cell cycle arrest, suggestive of dormancy or quiescence, as well as methotrexate chemoresistance (37). Moreover, high Mer expression in t(1;19) leukemia cells increase CNS involvement in murine xenografts and correlated with CNS leukemia at diagnosis in leukemia patients. Finally, Mer kinase inhibitors have been developed and could represent a novel therapy in leukemia patients with the t(1;19) translocation and high Mer expression (69).

### Influence of IL-15 on CNS Leukemia

As described in the prior section, the cytokine IL-15 has been implicated in CNS leukemia. In addition to upregulating genes in leukemia cells implicated in CNS trafficking, IL-15 also enhances leukemia proliferation through an effect on the Raf/Ras/ERK signaling pathway (60). This stimulation of leukemia growth was maximal under conditions of low or no serum supplementation, which the authors speculate may mimic the low-protein composition of CSF (60). Further supporting this possibility, high levels of IL-15 have been detected in the CSF and serum of patients with neuro-inflammatory disorders (70). The ability of IL-15 to regulate NK cell development, survival, and activation may provide another, indirect mechanism by which IL-15 influences CNS leukemia. NK cells are a component of the innate immune system with an important role in cancer immune surveillance (71). Frishman-Levy et al. used murine leukemia models and xenografts to show that expression of IL-15 by leukemia cells is associated with the activation of NK cells (72). However, while activated NK cells attenuated the growth of leukemia cells in the periphery *via* a NKG2D receptor-mediated mechanism, NK cells fail to effectively enter the CNS and, as a result, poorly control CNS leukemia. While this mechanism has yet to be demonstrated in patients, analysis of BM samples from pediatric leukemia patients showed high levels of the NKG2D receptor in infiltrating NK cells in patients with CNS leukemia (72).

### The CNS Niche Influences Leukemia Gene Expression Profiles

Further supporting an important role for the CNS microenvironment in leukemia biology, it has recently been shown that the CNS niche imparts unique and functionally important gene expression changes in both leukemia cells lines and primary xenografts (36, 73). In these experiments, human pre-B leukemia cells isolated from the BM and CNS microenvironments of the same mice were subjected to gene expression profiling analyses. Gene set enrichment analyses and functional annotation of the differentially expressed genes revealed that the genes were involved in multiple pathways important for cancer biology, including MAPK, RAS, apoptosis, as well as adaptation to hypoxia with enhanced quiescence and downregulation of oxidative phosphorylation. Additionally, genes dysregulated in leukemia cells isolated from the CNS were shown to be functionally important to leukemia biology. One study demonstrated that upregulation of the gene PBX1 in leukemia cells in the CNS microenvironment conferred enhanced leukemia chemoresistance and self-renewal properties (36). The other study showed that targeting VEGFA, one of the most upregulated genes in CNS-derived leukemia cells, with the VEGF neutralizing antibody bevacizumab reduced the extent of leukemia involvement in the CNS of mice (73). Furthermore, elevated CSF levels of VEGFA have been identified in patients with CNS leukemia (74). The authors speculate that VEGFA may further enhance migration of leukemia cells into the CNS through its effects on increasing endothelial, and potentially blood–brain, permeability. Together, these data support the proposition that the CNS provides a unique leukemia niche that influences leukemia biology.

Complementing these xenotransplantation approaches, gene expression profiling of leukemia cells isolated from the BM of high risk pediatric pre-B cell leukemia patients identified genes and pathways, including WNT, JAK, NF-κB, and B-cell receptor signaling, which distinguish patients with varying extents of CNS leukemia (CNS1-3) at the time of diagnosis (75). Although this work identifies genes and pathways that may either increase CNS homing or facilitate leukemia survival in the CNS, it does not identify the effects of the CNS niche on the leukemia transcriptome, as it utilized BM samples. In an attempt to define the effects of the CNS niche on leukemia cells, van der Velden et al. recently described a unique gene expression pattern in pre-B cell precursor ALL cells isolated from the CSF of patients with isolated CNS relapse when compared with leukemia cells isolated from the BM of patients at diagnosis (76). Moreover, for 5/8 patients with isolated CNS relapse data, they also had gene expression data from the patients’ leukemia cells isolated from the BM at time of diagnosis. However, while the samples were paired for patients, they were obtained at different times (i.e., diagnosis and CNS relapse). Unsupervised clustering analysis showed that the CSF-derived ALL samples were transcriptionally distinct from the BM ALL samples. Pathway analyses of the differentially expressed genes showed enrichment of genes involved in cellular development, cell death/survival, and several signaling pathways, including JAK/STAT and MAPK. Finally, a subpopulation of pre-B ALL cells with a “CNS leukemia profile” (*SCD* gene positive and increased *SPP1* gene expression) was identified in the BM of patients that later developed an isolated CNS relapse. In contrast, this population was low (<1%) or absent in all other patients. Moreover, the lack of a correlation between this leukemia subpopulation and morphologic CNS involvement at diagnosis raises the possibility that these leukemia genes and pathways may provide a survival advantage to the leukemia cells residing in the CNS niche rather than enhancing leukemia trafficking to the CNS. However, it is possible that more sensitive approaches for detecting CNS leukemia (PCR, flow cytometry) would have detected CNS disease in patients with a “CNS leukemia profile” at diagnosis and that these genes are important for trafficking to the CNS as well.

## Conclusion

Developing more effective therapies for CNS leukemia is crucial to long-term survival and quality of life for ALL patients. Given the importance of the microenvironment in many aspects of leukemia biology, more effective and less toxic therapies will likely only be realized through a better understanding of the effects of the CNS on critical aspects of leukemia biology. Since the BM and CNS are unique niches, research from the BM will not uniformly translate to the CNS leukemia niche. Supporting this, it has been shown that the CNS niche, relative to the BM, imparts unique effects on the leukemia proteome and transcriptome that influence important aspects of leukemia biology including chemoresistance (35, 36, 73). Furthermore, the complex role of the BM niche in leukemia and hematopoiesis suggests that much remains to be learned about role of the CNS niche in leukemia. For example, distinct niches within the BM, such as the endosteal and perivascular, uniquely influence normal hematopoiesis as well as leukemia biology. Accordingly, further defining at a cellular and molecular level, the components of the CNS that harbor and support chemoresistant leukemia cells will provide a foundation for experiments aimed at understanding and targeting the mechanisms by which the CNS niche influences leukemia biology. Similarly, more comprehensive analyses of the leukemia transcriptome, genome, and proteome in the CNS microenvironment will provide a more detailed understanding of the role of the CNS niche in leukemia as well as new insight into CNS relapse and leukemia biology. Finally, we anticipate this area of investigation will ultimately enhance both the durability and quality of the cure for ALL patients by identifying leukemia cell vulnerabilities unique to the CNS niche or approaches for disrupting the CNS niche (Figure 1C). For example, based on our current therapeutic arsenal and understanding of the genes, pathways, and molecules implicated in the pathophysiology of CNS leukemia (Table 1), potential approaches for augmenting CNS-directed therapy include (i) neutralizing or blocking antibodies directed against VEGF (bevacizumab), CCR7, or other adhesion molecules, (ii) Mer tyrosine kinase inhibitors in the setting of a t(1;19), or (iii) metabolic/mitochondrial modulation.

## Author Contributions

NG and PG both contributed to the conception, writing, and editing of this review.

## Conflict of Interest Statement

The authors declare that the research was conducted in the absence of any commercial or financial relationships that could be construed as a potential conflict of interest.
